# Dipeptidyl peptidase IV (DPPIV/CD26) inhibition does not improve engraftment of unfractionated syngeneic or allogeneic bone marrow after nonmyeloablative conditioning

**DOI:** 10.1016/j.exphem.2011.10.010

**Published:** 2012-02

**Authors:** Elisabeth Schwaiger, Christoph Klaus, Veerle Matheeussen, Ulrike Baranyi, Nina Pilat, Haley Ramsey, Stephan Korom, Ingrid De Meester, Thomas Wekerle

**Affiliations:** a Division of Transplantation, Department of Surgery, Vienna General Hospital, Medical University of Vienna, Austria; b Department of Pharmaceutical Sciences, Laboratory of Medical Biochemistry, University of Antwerp, Belgium; c Division of Thoracic Surgery, University Hospital Zurich, Switzerland

## Abstract

In order to develop minimally toxic bone marrow transplantation (BMT) protocols suitable for use in a wider range of indications, it is important to identify ways to enhance BM engraftment at a given level of recipient conditioning. CXCL12/stromal cell-derived factor-1α plays a crucial physiological role in homing of hematopoietic stem cells to BM. It is regulated by the ectopeptidase dipeptidyl peptidase IV (DPPIV; DPP4) known as CD26, which cleaves dipeptides from the N-terminus of polypeptide chains. Blocking DPPIV enzymatic activity had a beneficial effect on hematopoietic stem cell engraftment in various but very specific experimental settings. Here we investigated whether inhibition of DPPIV enzymatic activity through Diprotin A or sitagliptin (Januvia) improves BM engraftment in nonmyeloablative murine models of syngeneic (i.e., CD45-congenic) and allogeneic (i.e., Balb/c to B6) BMT (1 Gy total body irradiation, 10–15 × 10^6^ unseparated BM cells/mouse). Neither Diprotin A administered in vivo at the time of BMT and/or used for in vitro pretreatment of BM nor sitagliptin administered in vivo had a detectable effect on the level of multilineage chimerism (follow-up >20 weeks). Similarly, sitagliptin did not enhance chimerism after allogeneic BMT, even though DPPIV enzymatic activity measured in serum was profoundly inhibited (>98% inhibition at peak exposure). Our results provide evidence that DPPIV inhibition via Diprotin A or sitagliptin does not improve engraftment of unseparated BM in a nonmyeloablative BMT setting.

Allogeneic bone marrow transplantation (BMT) has therapeutic potential for a wide range of indications. Its clinical application remains limited mainly to the treatment of life-threatening diseases because of substantial toxicities associated with currently available BMT regimens.

Transplantation of donor BM to induce mixed hematopoietic chimerism is an attractive experimental approach to induce robust and lasting donor-specific tolerance in organ transplantation [1]. The clinical relevance of this tolerance strategy has recently been underscored by a pilot trial in which patients suffering from end-stage renal disease simultaneously received a kidney and BM graft from a human leukocyte antigen–mismatched living related donor [2,3]. Most recipients in this small study became operationally tolerant. However, the nonmyeloablative conditioning regimen was associated with substantial side effects, such as profound leukopenia, rendering this regimen virtually unacceptable in the routine organ transplantation setting. Therefore, less toxic BMT regimens achieving sufficient engraftment with reduced myelosuppressive conditioning still need to be developed to allow a more widespread application of this strategy [4].

In the experimental setting, less toxic mixed chimerism protocols have been generated gradually during the last several decades. The use of costimulatory blockers—some of which are already under clinical development as immunosuppressive drugs [5,6]—as part of BMT protocols has allowed us to further reduce conditioning substantially [7–11]. Short-course rapamycin [12,13] and therapeutic administration of regulatory T cells [1,14] have led to the most advanced murine minimum conditioning protocols. However, translation to nonhuman primate models has revealed that only transient chimerism is achieved with protocols that establish permanent chimerism in mice [15,16]. Development of adjunctive treatments capable of promoting engraftment of a given dose of BM at a certain level of recipient myelosuppression is a critical goal toward clinical translation of the mixed chimerism approach.

The chemoattractant stromal cell–derived factor-1 (CXCL12) binding to CXCR4 on hematopoietic stem cells (HSCs) plays an important role in regulating trafficking of HSCs to BM [17]. Dipeptidylpeptidase IV (DPPIV/CD26) is an ectopeptidase that cleaves stromal cell–derived factor-1 and thereby abrogates its chemotactic function [18] with the consequence of reduced homing of HSCs to their BM niches [19,20]. Specific inhibition of DPPIV/CD26 via Diprotin A, an enzymatic inhibitor consisting of three amino acids (Ile-Pro-Ile), enhanced BM engraftment in certain murine BMT models [21–25]. Notably, Christopherson et al. showed a benefit on engraftment when BM was incubated with Diprotin A before transplantation into myeloablated congenic recipients [20]. Combining in vivo with in vitro treatment with Diprotin A was found to further enhance its efficacy [24]. However, it remains undetermined whether DPPIV/CD26 inhibition promotes engraftment of unseparated BM in the nonmyeloablative mixed chimerism setting.

An immunosuppressive role of DPPIV inhibition has also been suggested in organ transplantation models (not involving BMT), as an irreversible inhibitor of DPPIV abrogated acute rejection in rat lung and heart transplantation models [26,27] and reduced ischemia/reperfusion injury [28]. This effect may be due to DPPIV/CD26-mediated truncation of mediators (such as cytokines and chemokines) [29,30], to a potential costimulatory function of CD26, or to so far unknown off-target effects of the inhibitor used [31–34].

We report that neither in vitro DPPIV/CD26 enzymatic inhibition of donor BM using Diprotin A nor additional systemic inhibition led to enhanced BM engraftment in a congenic murine model using 1 Gy total body irradiation (TBI) and conventional doses of BM. Moreover, we provide evidence that the clinically approved DPPIV inhibitor sitagliptin [35] completely blocked DPPIV/CD26 enzymatic activity in vivo, but nevertheless did not increase BM engraftment in either allogeneic or congenic models of nonmyeloablative BMT.

## Materials and methods

### Animals

Female C57BL/6NCrl (H-2^b^, CD45.2, denoted B6 herein), Balb/c (H-2^d^), and C3H/N (H-2^k^) were purchased from Charles River Laboratories (Sulzfeld, Germany), female B6.SJL-Ptprc^a^ Pep3^b^/BoyJ mice (H-2^b^, CD45.1, denoted CD45.1 B6 herein) were purchased from the Jackson Laboratory (Bar Harbour, ME, USA). All mice were housed under specific pathogen-free conditions and were used between 6 and 12 weeks of age. All experiments were approved by the local review board of the Medical University of Vienna, and were performed in accordance with national and international guidelines of laboratory animal care.

### BMT protocol

Treatment protocols per group are listed in Table 1. For the congenic setting (groups A through E) CD45.1 B6 were used as recipients and B6 (i.e., CD45.2) as donors. For the allogeneic setting (groups F and G) B6 were used as recipients, Balb/c as donors and C3H as third party. Recipients received 1 Gy TBI (day –1) and either 10 × 10^6^ or 15 × 10^6^ unseparated congenic or allogeneic BM cells (day 0), as indicated. Allogeneic recipients received costimulation blockade consisting of anti-CD154 monoclonal antibody (MR1, 1 mg, day 0), and hCTLA4Ig (abatacept, 0.5 mg, day 2) [12]. Indicated groups of mice received the following DPPIV inhibition regimens. BM in vitro pretreated with Diprotin A (group B); BM in vitro pretreated with Diprotin A plus in vivo recipient treatment with Diprotin A (group D); in vivo recipient treatment with sitagliptin (group E and G). Groups A, C, and F served as controls without DPPIV inhibition. Anti-CD154 monoclonal antibody was purchased from BioXCell (West Lebanon, NH, USA), hCTLA4Ig (abatacept) was generously provided by Bristol-Myers, Squibb Pharmaceuticals (Princeton, NJ, USA).

### DPPIV inhibition

Donor BM was harvested, washed, and resuspended in BM medium containing M199 (Sigma M 4530), DNAse (Sigma D-4527), gentamycin, and HEPES (1M) as described [12,36]. The control groups A, C, and F received BM in medium alone. In vitro pretreatment of BM with Diprotin A (groups B, D) was performed by incubating BM with a concentration of 5 mM Diprotin A for 15 min at room temperature [22] (group B) or for 15 min at 37°C [21] (group D). Subsequently, bone marrow cells (BMC) were washed in BM medium, counted, and resuspended. For in vivo recipient treatment with Diprotin A (group D), BMC were resuspended in 1 mL BM medium containing 4 μmol Diprotin A, which was injected intravenously and 100 μL phosphate-buffered saline containing 5 μmol Diprotin A injected subcutaneously every 12 hours for 3 days (days 0, 1, and 2). This daily dose of Diprotin A was chosen as it showed therapeutic effects in other murine models [37,38]. For in vivo recipient treatment with sitagliptin (groups E and G), sitagliptin tablets (100 mg) were suspended in 2 mL cold phosphate-buffered saline under aseptic conditions, resulting in 4 mg/80 μL. Four milligrams sitagliptin was administered by oral gavage every 12 hours on days 0 and 1 (group G) or on days 0, 1, and 2 (group E) post-BMT. Diprotin A was purchased from Sigma Aldrich and sitagliptin (Januvia) was kindly provided by Merck (Vienna, Austria).

### Assay for DPPIV activity

DPPIV enzymatic activity was assayed by using glycyl-prolyl-4-methoxy-β-naphthylamide (Gly-Pro-4-Me-β-NA) as fluorogenic substrate as described previously [39,40]. In a 96-well plate, 5 μL serum samples were mixed with 0.5 mM Gly-Pro-4-Me-β-NA in 50 mM Tris buffer (pH 8.3) in a final volume of 110 μL. DPPIV activity was determined kinetically during 5 min at 37°C by measuring the velocities of 4-Me-β-NA release (λ_ex_ = 340 nm, λ_em_ = 430 nm) from the substrate using an Infinite 200 (Tecan Group Ltd., Switzerland) (all reagents were purchased from Sigma-Aldrich). Fluorescence intensity was related to a 4-Me-β-NA standard curve. The reversibility of the inhibitors in the serum samples and the dilution of these samples in the assay make it necessary to create a calibration curve with known concentrations of the inhibitors in murine serum to estimate the percentage in vivo inhibition of DPPIV enzymatic activity in the serum. Percentage inhibition was calculated by comparing DPPIV enzymatic activity of treated mice to control mice, which were not enzymatically inhibited (defined as 100% activity). In order to limit the number of blood draws per mouse, groups were split in two and blood was taken only once a day for each mouse (either at 2 hours or 12 hours post–DPPIV inhibitor administration).

### Flow cytometric analysis

Two-color flow cytometric analysis was used to distinguish donor and recipient cells of particular lineages by staining with fluorescein isothiocyanate–conjugated antibodies against CD4, CD8, B220, MAC-1, and biotinylated CD45.2 or 34-2-12 (H-2D^d^, detected with phycoerythrin-streptavidin) and irrelevant isotype controls [12,36]. Propidium iodide staining was used to exclude dead cells. The net percentage of CD45.2^+^ or 34-2-12^+^ live cells among different cell lineages was calculated. Mice were considered chimeric if they demonstrated at least 2% of donor cells within the myeloid lineage plus at least one lymphoid lineage. Surface staining was performed according to standard procedures and flow cytometric analysis was done on a Coulter Cytomics FC500. CXP software (Coulter, Vienna, Austria) was used for acquisition and analysis. Antibodies were purchased from Becton Dickinson (San Diego, CA, USA).

### Skin grafting

Full-thickness tail skin from Balb/c mice and fully mismatched C3H (third party) was grafted 2 to 8 weeks after allogeneic BMT and visually inspected thereafter at short intervals. Grafts were considered rejected when <10% remained viable.

### Statistics

A two-sided Student’s *t* test was used to compare chimerism levels between the groups. Skin graft survival was calculated according to the Kaplan–Meier product limit method and compared between groups using the log-rank test.

## Results

### Inhibition of DPPIV with Diprotin A does not improve engraftment of unseparated congenic BM after nonmyeloablative conditioning

**Figure 1:**
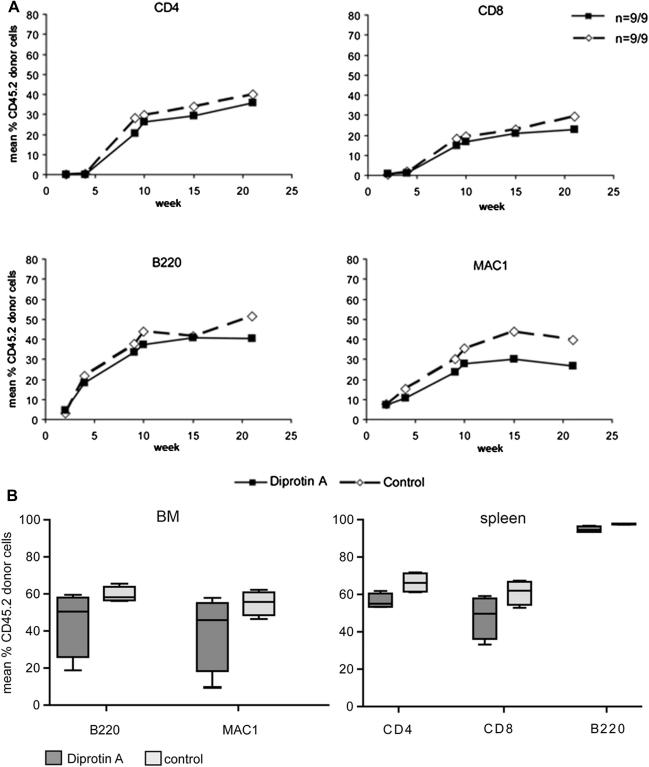
Chimerism following transplantation of congenic BMCs pretreated in vitro with Diprotin A. Recipient mice were conditioned with 1 Gy TBI and received 15 × 10^6^ congenic CD45.2 BMCs (n = 9/control group A, n = 9/Diprotin A–treated group B). BMCs of group B were treated in vitro with 5 mM Diprotin A before transplantation. The levels of chimerism in blood over time (**A**), was determined by flow cytometry and is presented as means for Diprotin A–treated (squares) and untreated (dotted line with diamonds) groups. In (**B**) chimerism in BM and spleen at the end of follow-up is depicted in box and whisker plots. No significant differences were noted between both groups.

To investigate the effect of DPPIV inhibition on the engraftment of unseparated BM in the absence of alloreactivity, we first used a CD45.2 → CD45.1 congenic donor-recipient combination [36,41]. CD45.1 B6 mice conditioned with 1 Gy TBI received 15 × 10^6^ unseparated CD45.2 BMCs that were or were not pretreated in vitro with the DPPIV inhibitor Diprotin A (n = 9/group) [22]. Multilineage chimerism was followed in blood by flow cytometry. Lasting chimerism developed in all mice in both groups. During a period of 21 weeks post-BMT, chimerism levels were comparable between Diprotin A–pretreated (group B) and nontreated (group A) groups in all tested lineages at all analyzed time points (note: treated and nontreated groups were done in parallel within one experiment to allow optimal comparability) (Fig. 1A). At the end of follow-up, mean percentages (±standard deviation) of chimerism were 35.7% (±7.0%) vs 39.9% (±7.7%) among CD4 cells, 23.0% (±4.7%) vs 29.3% (±9.1%) among CD8 cells, 40.6% (±10.7%) vs 51.3% (±13.4%) among B cells, and 26.7% (±10.2%) vs 39.5% (±14.9%) among myeloid cells (Diprotin A pretreatment vs control group; *p* = NS for all lineages). Similarly, there were no differences in chimerism levels between groups in BM and spleen (*p* = NS, Fig. 1B).

**Figure 2:**
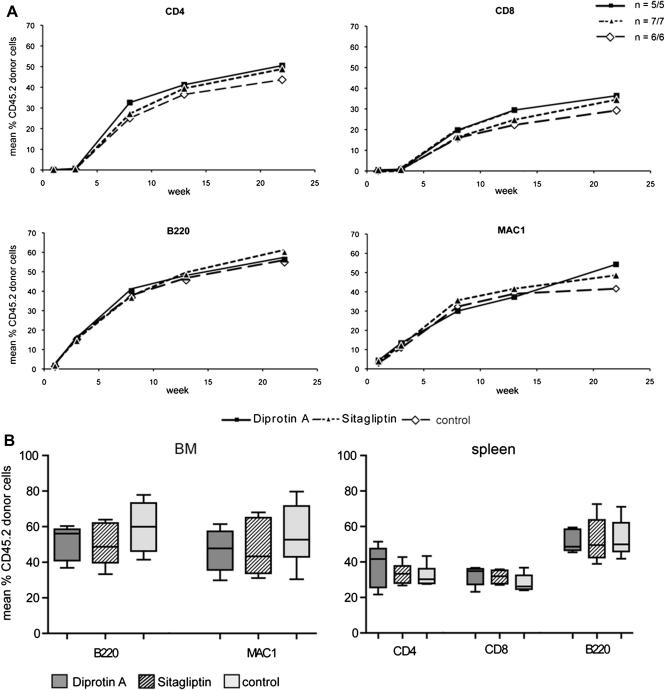
Chimerism after transplantation of congenic BMCs after combined treatment with Diprotin A in vitro and in vivo and after in vivo treatment with sitagliptin. Recipient mice were conditioned with 1 Gy TBI and received 10 × 10^6^ congenic CD45.2 BMCs (n = 6/control group C, n = 5/Diprotin A–treated group D, n = 7/sitagliptin-treated group E). In group D, BMC were treated in vitro with 5 mM Diprotin A before transplantation and, in addition, recipients were treated in vivo with Diprotin A (4 μmol IV day 0 and 5 μmol Diprotin A subcutaneously every 12 hours for 3 days). Recipients of group E were treated with sitagliptin orally. Levels of chimerism in blood over time (**A**) were determined by flow cytometry and are presented as means for Diprotin A–treated (squares), sitagliptin-treated (dotted line with triangle) and untreated (dotted line with diamonds) groups. In (**B**) chimerism in BM and spleen at the end of follow-up is depicted in box and whisker plots. No significant differences were noted between both groups.

Because a beneficial effect has been described when in vitro Diprotin A pretreatment of donor BM was combined with in vivo recipient treatment with Diprotin A [24], we tested whether such a combination regimen would impact engraftment in the nonmyeloablative congenic setting (group D). In addition to in vitro pretreatment of the BM, Diprotin A was injected in vivo together with the BM and every 12 hours thereafter for 3 days post-BMT (n = 5). Again, chimerism levels were similar with (group D) and without (group C) DPPIV inhibition at all tested time points (follow-up 22 weeks; *p* = NS for all time points) (Fig. 2A). At the end of follow-up, mean percentages of chimerism were 50.5% (±5.8%) vs 43.7% (±6.4%) among CD4 cells, 36.4% (±4.7%) vs 29.3% (±6.5%) among CD8 cells, 56.2% (±6.7%) vs 54.9% (±9.5%) among B cells, and 54.2% (±10.0%) vs 41.6% (±11.5%) among myeloid cells. Chimerism levels in BM and spleen were also comparable among groups.

Collectively, these experiments demonstrate that DPPIV inhibition with Diprotin A does not lead to a detectable improvement of the engraftment of unseparated congenic BM in nonmyeloablatively conditioned recipients.

### Sitagliptin inhibits DPPIV enzymatic activity more effectively than Diprotin A

To assess whether the failure to detect an engraftment effect is due to insufficient DPPIV inhibition achieved with in vivo Diprotin A treatment, serum DPPIV activity was measured.

**Figure 3:**
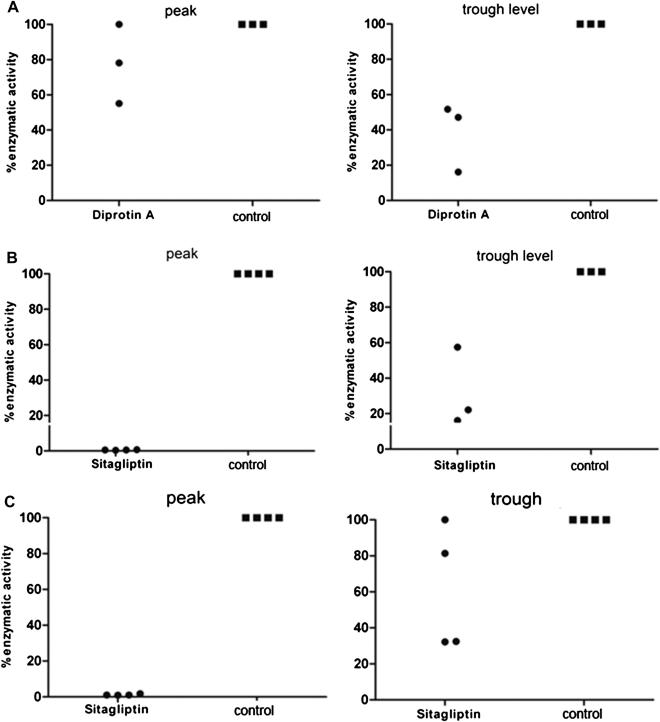
DPPIV enzymatic activity in serum after in vivo inhibition with Diprotin A or sitagliptin. DPPIV enzymatic activity in serum was measured 2 (peak) and 12 hours (trough) after in vivo treatment with Diprotin A (group D) or sitagliptin (group E). DPPIV enzymatic activity at peak and trough exposure is depicted for Diprotin A– (**A**) and sitagliptin-treated [(**B**) congenic, (**C**) allogeneic model] groups (n = 3–4 randomly selected mice per group, congenic BMT).

At peak exposure (2 hours after administration of Diprotin A, group D), DPPIV serum activity decreased to 78.1% and 55.1% in two (randomly selected) mice, whereas DPPIV activity remained essentially unchanged in one mouse (Fig. 3A). At the time of trough exposure (12 hours postadministration), enzymatic activity declined to 51.7%, 47.1%, and 16.1% in three remaining mice of the group (Fig. 3A). Thus, although Diprotin A inhibits DPPIV activity, inhibition is only moderate.

Sitagliptin (Januvia) is a specific DPPIV inhibitor that has recently been approved for the treatment of type 2 diabetes [42]. We hypothesized that a more complete (and clinically relevant) inhibition of DPPIV may be achieved with this drug. Sitagliptin has a half-life of about 12 to 14 hours [43] and is administered once daily at a dose of 100 mg (i.e., roughly 1.3 mg/kg) in the clinical setting of type 2 diabetes. We measured DPPIV activity in serum of mice treated with 4 mg sitagliptin orally every 12 hours (160 mg/kg twice daily) (group E). Complete inhibition of DPPIV enzymatic activity at peak exposure (2 hours postadministration) was observed in all four tested mice (0.7%, 0.4%, 0.5%, and 0.5% enzymatic activity) (Fig. 3B). At trough level 12 hours after the last dose, enzymatic DPPIV activity decreased to a mean of 31.9% (i.e., 68% inhibition) (16.2%, 22.1%, 57.4% in the remaining three mice of the group) (Fig. 3B). Thus, in vivo treatment with sitagliptin leads to more effective inhibition of DPPIV activity at peak exposure than Diprotin A. However, with neither compound, a complete DPPIV inhibition for the entire treatment period was achieved, as moderate enzymatic activity at trough levels could still be observed.

### Despite superior DPPIV inhibition, sitagliptin does not improve engraftment of unseparated congenic BMT after nonmyeloablative conditioning

To assess whether sitagliptin affects engraftment, we followed hematopoietic chimerism in sitagliptin-treated recipients of unseparated congenic BM (n = 7, group E). Again, no enhanced engraftment was detected on DPPIV inhibition (*p* = NS at all time points). Twenty-two weeks post-BMT mean chimerism levels were 48.8% (±5.7%) vs 43.7% (±6.4%) among CD4 cells, 34.5% (±3.0%) vs 29.3% (±6.5%) among CD8 cells, 60.1% (±9.8%) vs 54.9% (±9.5%) among B cells, and 48.6% (±11.1%) vs 41.6% (±11.5%) among myeloid cells (treated group E vs control group C) (Fig. 2A). Chimerism levels in BM and spleen (Fig. 2B) were also comparable (*p* = NS). Thus, with the more specific, clinically approved DPPIV inhibitor sitagliptin, no beneficial effect was detectable on the engraftment of congenic BM in nonmyeloablated recipient mice.

### Sitagliptin does not improve engraftment in a mixed chimerism model of allogeneic BMT with nonmyeloablative conditioning and costimulation blockade

DPPIV inhibition was shown to affect alloreactivity in models of heart and lung transplantation [26,27]. As little is known, however, about the effect of DPPIV inhibitors in allogeneic BMT, we tested whether sitagliptin may improve engraftment in an allogeneic mixed chimerism model of limiting conditioning. B6 mice conditioned with 1 Gy TBI (day –1), were transplanted with 15 × 10^6^ fully mismatched Balb/c BMCs (day 0), and treated with anti-CD154 monoclonal antibody (day 0) and CTLA4Ig (day +2), as described previously [12]. This BMT regimen is insufficient to induce reliable chimerism (and tolerance) by itself, but chimerism and tolerance can be achieved through adjunctive treatments, such as rapamycin [12]. One group was treated with sitagliptin orally (group G, n = 6) and was compared to one left untreated (group F, n = 6).

**Figure 4:**
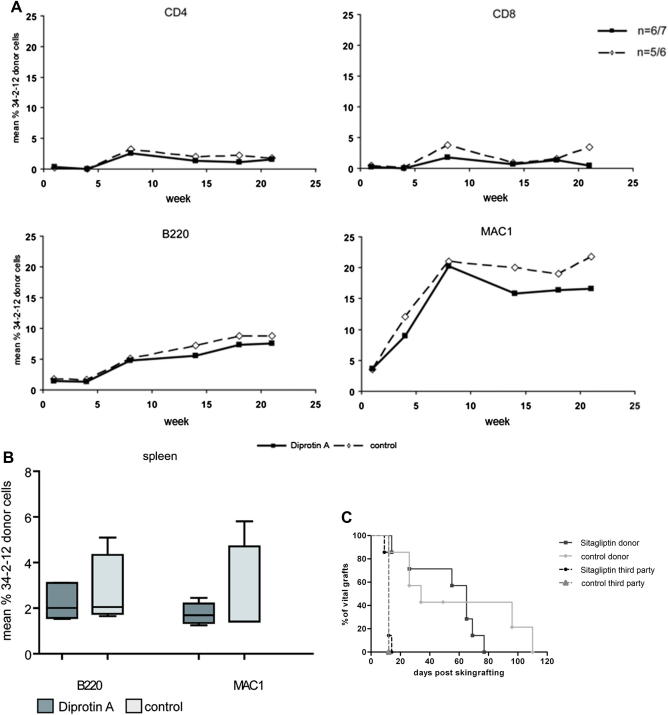
Chimerism and skin graft survival following transplantation of allogeneic BMCs after in vivo treatment with sitagliptin. Recipient mice were transplanted with 15 × 10^6^ allogeneic BMCs after 1 Gy TBI (day –1) and costimulation blockade consisting of anti CD-154 monoclonal antibody (day 0) and CTLA4Ig (day 2). Four milligrams sitagliptin per mouse were administered twice a day (day 0–2). (**A**) Mean percent of blood chimerism among different cell lineages over time are depicted for sitagliptin-treated (bold lines with squares) and untreated (dotted lines with triangles) groups. No significant differences were noted between both groups. (**B**) Chimerism in spleen was similar in both groups at the end of follow-up. (**C**) Approximately 8 weeks post-BMT, mice were grafted with donor and third-party skin. Skin graft survival was comparable in both groups (*p* = 0.5).

Again, sitagliptin nearly completely inhibited DPPIV enzymatic activity after 2 hours (0.9%, 1.0%, 1.0%, 1.8%) and activity remained substantially decreased in 2 of 4 mice (32.2%, 32.5%, 81.4%, >85%) after 12 hours (Fig. 3C). As expected, without sitagliptin, mean chimerism levels were low with varying individual levels and several mice had no detectable T-cell chimerism at the end of follow-up (Fig. 4A). Sitagliptin treatment, however, did not improve chimerism levels. Chimeric mice (six of seven chimeras in the sitagliptin-treated group vs five of six in the control group) showed chimerism levels of 1.6% (±1.1%) vs 1.8% (±2.5%) among CD4 cells, 0.4% (±0.6%) vs 3.5% (±3.1%) among CD8 cells, 7.5% (±3.8%) vs 8.8% (±4.1%) among B cells, and 16.6% (±12.5%) vs 21.8% (±10.3%) among myeloid cells for each lineage (*p* = NS). Similarly, chimerism levels and rates were also comparable between groups in spleen (Fig. 4B).

Donor and third-party skin was transplanted to assess donor-specific tolerance. Third-party grafts were promptly rejected in both groups. Donor skin graft survival was significantly prolonged in both groups but tolerance was not achieved, consistent with the poor T-cell chimerism (*p* = 0.5 sitagliptin-treated vs untreated recipients) [8,44].

Taken together, these results indicate that sitagliptin does not improve engraftment of allogeneic BM under limiting recipient conditioning and consequently does not improve tolerance induction in this setting.

## Discussion

Induction of donor-specific transplantation tolerance through mixed chimerism would be a potential new indication for allogeneic BMT. During the last 2 decades, the toxicity of the recipient conditioning was gradually reduced in murine chimerism models [1]. Nevertheless cytotoxic conditioning used to achieve engraftment of clinically feasible numbers of BMCs keeps impeding clinical translation of such protocols. Minimally toxic BMT regimens would also be of interest to allow other potential indications of BMT [9].

Inhibition or genetic ablation of DPPIV/CD26 showed promising engraftment-promoting effects in several murine models especially when limited numbers of donor cells were used [25]. DPPIV enzymatic activity inhibition was effective in congenic (CD45-congenic) [20,38], xenogeneic (human cells into nonobese diabetic severe combined immune-deficient recipients) [23,25], and allogeneic (major histocompatibility complex–mismatched in utero transplantation) [22] systems. Engraftment-enhancing effects of DPPIV inhibition were not only observed with separated (Sca1^+^lin^−^ murine BMCs or CD34^+^ human mobilized peripheral blood/cord blood cells, respectively) [20,24,25], but also with unseparated populations [20,22,38]. We therefore chose nonmyeloablative protocols using unseparated BMCs for evaluating DPPIV enzymatic inhibition, as such systems are commonly used in the clinical setting (but to the best of our knowledge have not been studied in the DPPIV inhibition context before). In addition, we targeted DPPIV in a fully allogeneic model, which would be similar to the clinical situation of tolerance induction in organ transplantation. In these settings, we did not detect a significant effect of DPPIV enzymatic activity inhibition on BM engraftment (despite using Diprotin A treatment schedules proven effective in other settings).

In order to dissect whether a potential effect of DPPIV inhibition is due to modulating alloreactivity or HSC engraftment, we used two established models of nonmyeloablative BMT. The CD45.2 → CD45.1 congenic model is almost free of relevant immunological barriers [36,41]. In the 1-Gy allogeneic protocol, immunosuppression—through rapamycin [12] or administration of T regulatory cells [45]—leads to engraftment and lasting chimerism in this otherwise unsuccessful BMT protocol.

Several DPPIV inhibitors have recently been approved for treatment of type 2 diabetes. We therefore tested whether sitagliptin enhances the engraftment of congenic or allogeneic BM after nonmyeloablative TBI. As compared to Diprotin A, sitagliptin is both a more potent and specific DPPIV inhibitor [46] that would be of importance for translating experimental results to the clinical setting. Despite inhibition of DPPIV enzymatic serum activity, no beneficial effect of sitagliptin was detectable in our studies.

Maximum inhibition of DPPIV serum activity (measured 2 hours after in vivo administration) was more profound with sitagliptin—achieving almost 100% inhibition—than with Diprotin A (Fig. 3). A rapid recovery of DPPIV systemic activity after Diprotin A treatment was also noted by Christopherson and colleagues [20], but this transient inhibition was nevertheless sufficient to positively affect engraftment in the myeloablative setting. Kim and colleagues reported prolongation of islet graft survival with sitagliptin [47]. Sitagliptin was given in chow with a sitagliptin uptake of roughly 10 mg—48 mg per day resulting in 78% to 88% DPPIV inhibition. Notably, almost the full inhibitory effect was observed even with the lowest consumed dose of 10 mg (approximately 78% inhibition). In the clinical setting of type 2 diabetes, sitagliptin is administered once daily, as it has a half-life of about 12 to 14 hours [43]. We therefore reasoned that oral administration every 12 hours (at a dose of 160 mg/kg twice daily compared to the clinical dose of approximately 1.3 mg/kg/day) would result in sufficient drug exposure during the critical period of HSC engraftment. It seems unlikely that the degree or duration of DPPIV inhibition is responsible for the lack of a detectable engraftment effect in our models.

We think that several factors might be responsible for the lack of an effect of DPPIV inhibition in our present study. Models in which an engraftment-enhancing of DPPIV inhibition was found used either myeloablative conditioning or nonmyeloablative conditioning of immunodeficient recipients [20,21,23–25,38]. To the best of our knowledge, our studies are the first to investigate the effect of DPPIV inhibition on BM engraftment in nonmyeloablatively conditioned wild-type recipients. In the environment of this different conditioning regimen, DPPIV inhibition might be unable to affect engraftment. Furthermore, an engraftment-enhancing effect of DPPIV inhibition on allogeneic HSC has been shown so far only after myeloablative conditioning or in utero transplantation [21,22]. The potential effect on allogeneic BM engraftment after nonmyeloablative conditioning—which is of relevance for clinical BMT—has not been ascertained previously. Our results suggest that DPPIV inhibition is of limited therapeutic value in this setting. Besides, the current study is the first to investigate the use of costimulation blockade together with DPPIV inhibition. Although an interaction cannot be ruled out, such interference would be unable to explain the observed lack of an effect in the congenic system, in which no costimulation blockade was used.

In summary, although DPPIV enzymatic inhibition had been demonstrated to have engraftment-promoting effects in several specific models of HSC transplantation, our studies provide evidence that DPPIV inhibition with Diprotin A or with sitagliptin does not lead to improved engraftment of unseparated BM after nonmyeloablative recipient conditioning.

## Figures and Tables

**Table 1:** Experimental protocols

Group	TBI	HSCT (cells/mouse)	CB	Additional treatment		Mouse strain
A	1	15 × 10^6^ BMC	−	-		Congenic
B	1	15 × 10^6^ BMC	−	5 mM Diprotin A in vitro (15 min)		Congenic
C	1	10 × 10^6^ BMC	−	-		Congenic
D	1	10 × 10^6^ BMC	−	5 mM Diprotin A in vitro (15 min) & in vivo (4 μM Diprotin A with BM*iv* & 5 μM Diprotin A *sc* 2×/day)	(72h)	Congenic
E	1	10 × 10^6^ BMC	−	Sitagliptin in vivo (4 mg/mouse/2×/day)	(72h)	Congenic
F	1	15 × 10^6^ BMC	+	-		Allogenic
G	1	15 × 10^6^ BMC	+	Sitagliptin in vivo (4 mg/mouse/2×/day)	(48h)	Allogenic
